# A Three-Dimensional Engineered Artery Model for In Vitro Atherosclerosis Research

**DOI:** 10.1371/journal.pone.0079821

**Published:** 2013-11-14

**Authors:** Jérôme Robert, Benedikt Weber, Laura Frese, Maximilian Y. Emmert, Dörthe Schmidt, Arnold von Eckardstein, Lucia Rohrer, Simon P. Hoerstrup

**Affiliations:** 1 Swiss Center for Regenerative Medicine, University and University Hospital Zürich, Zürich, Switzerland; 2 Clinic for Cardiovascular Surgery and Department of Surgical Research, University Hospital Zürich, Zürich, Switzerland; 3 Institute of Clinical Chemistry, University Hospital Zürich, Zürich, Switzerland; 4 Zurich Centre of Integrated Human Physiology, University of Zürich, Zürich, Switzerland; 5 Department of Cardiology, Cardiovascular Center, University Hospital Zürich, Zürich, Switzerland; William Harvey Research Institute, Barts and The London School of Medicine and Dentistry, Queen Mary University of London, United Kingdom

## Abstract

The pathogenesis of atherosclerosis involves dysfunctions of vascular endothelial cells and smooth muscle cells as well as blood borne inflammatory cells such as monocyte-derived macrophages. In vitro experiments towards a better understanding of these dysfunctions are typically performed in two-dimensional cell culture systems. However, these models lack both the three-dimensional structure and the physiological pulsatile flow conditions of native arteries. We here describe the development and initial characterization of a tissue engineered artery equivalent, which is composed of human primary endothelial and smooth muscle cells and is exposed to flow in vitro. Histological analyses showed formation of a dense tissue composed of a tight monolayer of endothelial cells supported by a basement membrane and multiple smooth muscle cell layers. Both low (LDL) and high density lipoproteins (HDL) perfused through the artery equivalent were recovered both within endothelial cells and in the sub-endothelial intima. After activation of the endothelium with either tumour necrosis factor alpha (TNFα) or LDL, monocytes circulated through the model were found to adhere to the activated endothelium and to transmigrate into the intima. In conclusion, the described tissue engineered human artery equivalent model represents a significant step towards a relevant in vitro platform for the systematic assessment of pathogenic processes in atherosclerosis independently of any systemic factors.

## Introduction

Atherosclerosis is responsible for a substantial global disease load and remains a major cause of morbidity and mortality worldwide. One of the first events in the pathogenesis of atherosclerosis is the insudation of cholesterol-rich low density lipoprotein (LDL) into the innermost intimal layer of the arterial wall where they are bound by proteoglycans and eventually modified [1]. In parallel and further enhanced by the retained lipoproteins, the activated endothelium binds immune cells such as monocytes, which subsequently transmigrate the endothelium. Within the arterial intima the monocytes differentiate into macrophages which upon receptor mediated uptake of LDL are transformed into foam cells. They form the early “fatty streak” lesion [2]. The inflammatory reaction of macrophage foam cells but also other immigrating immune cells induce the progression to more complicated lesions and ultimately vulnerable plaques, which upon rupture or erosion elicit the acute complications of atherosclerosis such as acute coronary events. High density lipoproteins (HDL) counteract several of these pro-atherogenic activities by various mechanisms. The classical potentially anti-atherogenic property is related to its ability to remove cholesterol from the macrophage foam cells within the arterial wall and mediate the transport to the liver for excretion into the bile [3]. To do this HDL must also cross the endothelial barrier in order to get access to the cholesterol-loaded macrophages [3].

In addition to various in vivo models, notably genetically modified mice, [4] cell culture experiments have been valuable tools to unravel the above summarized pathomechanisms of atherosclerosis. However - so far - most of the in vitro experiments are performed using single types of cells grown on regular static plastic dishes. Importantly, these studies are associated with significant limitations, as they do not account for the complexity of the native artery environment with all the cell-cell and/or cell-matrix interactions. To address these limitations, several laboratories developed co-culture models of endothelial cells and smooth muscle cells [5]–[7]. However, also these studies were limited by the unphysiological attachment of cells to plastic dishes or trans-well membranes and co-culture times which were too short for the development of typical vascular cell-extracellular matrix interactions. In the attempt to address these limitations, Dorweiler et al. developed a long-term co-culture setup on fibrin gels [8]. In this model they demonstrated for the first time the feasibility to analyse in vitro the accumulation of LDL and immunocytes in a sub-endothelial matrix [8], [9]. However, also this static and non-dynamic co-culture system is limited by the lack of both the circular structure of arteries and the physiological vascular hemodynamic situation characterized by flow and shear-stress.

In recent years, several laboratories fabricated tissue engineered artery equivalents under pulsatile, native-like flow conditions for the therapeutic and regenerative repair of congenital and acquired malformations [10]–[13]. These bioengineered autologous cell-based constructs were also successfully implanted into large animal models and revealed native-like behaviour and development up to 240 weeks in vivo [14]. In the present study we used human vascular cells to engineer a 3D artery model mimicking the structural as well as functional characteristics of a native artery. This native-analogous bioengineered artery model was used to study initial events in atherosclerosis, namely the accumulation of LDL and HDL in the intima as well as the binding and transmigration of monocytes under dynamic pulsatile flow conditions.

## Methods

### Isolation of Umbilical cord cells

Mature vascular cells were isolated from human umbilical cord vessels. All experiments were performed under Zurich cantonal ethical permission and written informed consent was obtained from all patients [KEK-2009-95]. Human umbilical vein endothelial cells (HUVECs) and human umbilical cord derived myofibroblasts (UCMFBs) were isolated and characterized as previously described [10], [14]. In brief, HUVECs were isolated using the collagenase instillation technique. For this, the umbilical cord vessels were incubated in collagenase (2 mg/ml; Collagenase A; Roche Diagnostics GmbH.) dissolved in serum-free medium (EBM™; LONZA Inc., Switzerland). After 20 min of incubation, the cell suspension was centrifuged and isolated cells were expanded in endothelial growth medium (EGM™-2) (LONZA Inc., Switzerland; supplemented with vascular endothelial growth factor (VEGF), human recombinant insulin-like growth factor-1 (hrIGF-1), human epidermal growth factor (hEGF), gentamycin, amphotericin-B, hydrocortisone, ascorbic acid, heparin, and 2% fetal bovine serum (FBS)). For the isolation of UCMFBs the remaining vessels were minced into small pieces (∼2–3 mm) and incubated without medium under the sterile laminar flow for 25–30 min to ensure physical attachment of the minced pieces. Subsequently, advanced Dulbecco’s Modified Eagle Medium (DMEM) medium (Invitrogen Corp., USA; supplemented with 10% FBS; 0.05% Penicillin/Streptomycin, 0.02% Fungizone and 1% L-glutamine) was carefully added to the minced vessel pieces and the adherent myofibroblastic cells were expanded up to passage 6.

### In vitro fabrication of tissue engineered vascular grafts

Biodegradable tubular scaffold matrices (length 4 cm and inner diameter 0.6 cm) were fabricated as described previously[10]. Briefly, non-woven polyglycolic-acid meshes (PGA; Cellon, Luxembourg) were dip-coated with poly-4-hydroxybutyrate (P4HB; TEPHA Inc., USA) by dipping it into a 1.75% (w/w) solution of P4HB/tetrahydrofuran solution (Sigma Aldrich). Vascular shape was obtained by heat application and a welding technique before external coating with 10% P4HB/tetrahydrofuran solution. After sterilization with 70% ethanol (30 min) followed by 3 washes with PBS or by ethylene oxide exposition, PGA-P4HB composite scaffolds were pre-incubated in DMEM medium overnight before cells seeding. UCMFBs (3–4×10^6^ cells/cm^2^) were seeded in the inner surfaces of the vascular scaffold using fibrin (Sigma Aldrich) as a cell carrier [15]. After a short static incubation period (3 days), vascular constructs were exposed to dynamic conditioning in a flow bioreactor system for 14 days. The flow of nutrient medium (DMEM with 10% FBS; 0.05% Penicillin/Streptomycin, 0.02% Fungizone, 1% L-glutamin and 1.5 mM L- ascorbic acid) was directed through their inner lumen of the bioreactor circulation loop, exposing the seeded constructs to an increasing flow over time (from 5 to 10 mL/min). Vascular grafts were then endotheliazed with HUVECs (1.5×10^6^ cells/cm^2^) and cultivated first in static condition for 5 days in EGM™-2 medium with supplements as described above. After the static phase, vascular grafts were placed back in the bioreactor for 14 additional days with increasing medium flow (from 3 to 10 mL/min).

### Isolation and labeling of LDL and HDL

Human LDL (1.006<d<1.063 kg/L) and HDL (1.063<d<1.21 kg/L) were isolated from fresh normolipidemic plasmas of blood donor by sequential gradient ultracentrifugation [16]. The purity of the lipoprotein preparation was verified by sodium dodecyl sulfate- polyacrylamide gel electrophoresis (SDS-PAGE) in order to assure no cross or albumin contamination. Freshly isolated LDL or HDL was fluorescently labeled with atto-633 (Invitrogen) following manufacturer instructions. Fluorescent LDL (20 µg/ml in EGM™-2 medium supplemented as described previously) was injected in the circulatory loop of the bioreactor in the absence or presence of a 40 folds excess (0.8 mg/ml) of non-labeled LDL. After 2.5 or 24 hours vascular grafts were removed and processed directly using confocal microscopy (CLSM SP5, Leica) or frozen in embedding matrix (O.C.T., Biosystem) for further analyses. Fluorescently labeled HDL (25 µg/ml in EGM™2 medium supplemented as described previously) was injected into the circulatory loop of the bioreactor system or injected into the medium adherently cultured cells. After 4 or 24 hours tissue engineered vascular grafts were removed and further processed as described for LDL.

### Isolation and labeling of monocytes

Monocytes were isolated from healthy donors by centrifugation on a continuous density gradient (Percoll™; Amersham bioscience, GE health care) as described previously [17]. Freshly isolated monocytes were fluorescently labeled with 10 µM of SNARF®-1 carboxylic acid (Invitrogen) following the manufacturer’s instructions. 1×10^6^ monocytes per mL were injected into the circulation loop of the bioreactor in EGM™-2 medium supplemented as described above but with 2% of FBS.

### Histology and immunohistochemistry of tissue engineered arteries

For histological characterization bioengineered grafts were fixed in 4%-paraformaldehyde (PFA), dehydrated through a series of graded ethanol, embedded in paraffin and sectioned at 7 µm thickness. The sections were deparaffinized, rehydrated through a graded ethanol series. The tissue sections were stained using haematoxylin & eosin, haematoxylin & sudan and Masson-trichrome. Immunohistochemistry analyses were performed with antibodies specific for CD31 (clone JC/70A, DakoCytomation, Glostrup, Denmark), α-smooth muscle actin (clone 1A4, Sigma) and collagen IV (Quartett, Germany) using the Vantana Benchmark automated staining system (Ventana Medicals Systems, Tucso,AZ) as previously described [10].

### Biochemical extracellular matrix analysis

Representative tissue samples were lyophilized and analyzed by biochemical assays for total DNA content as an indicator for cell number, hydroxyproline (HYP) content as an indicator for collagen, as well as glycosaminoglycans (GAG) content. For measuring the DNA amount, the Hoechst dye method [18] was used with a standard curve prepared from calf thymus DNA (Sigma Chemical Co., USA). The GAG content was determined using a modified version of the protocol described by *Farndale* et al. [19] and a standard curve prepared from chondroitin sulphate (Sigma Chemical Co., USA). HYP was determined using a modified version of the protocol provided by *Huszar* et al. [20]. Native control tissues from human aorta were included and all values are presented relative to these native values (% of native).

### Confocal microscopy

Bioengineered grafts were first washed twice in PBS and then fixed in 3.75% of PFA for 30 minutes at room temperature. Following a second series of PBS washing phases (3x), grafts were mounted in 0.1 M Tris-HCL, pH 9.5, and glycerol (3:7) containing 50 mg/ml of n-propyl gallats as anti-fading reagent [21] and 1 ng/ml of 4',6-diamidino-2-phenylindole (DAPI) to visualize the nuclei. Analyses of the engineered graft samples were performed on an inverted confocal microscopy.

### Preparation of the cryopreserve sections

Bioengineered grafts were first washed twice in PBS and then cryopreserved in embedding matrix (O.C.T.). The grafts were further processed on cryotome generating cuts with 12 µm thickness. The cuts were stored at –80°C until further processing. Cuts were rehydrated in PBS for 3×10 minutes before fixing in 3.75% of PFA for 20 minutes at room temperature. After three PBS washes cuts were blocked for 30 minutes in 5% donkey serum in PBS. For immunofluorescence staining, the cuts were incubated overnight at 4 °C with specific antibodies against ZO1 (33-9100, Invitrogen), ABCG1 (HG5, Santa Cruz), SR-BI (NB 400-101 E4, Novus) or EL (NB 400-111, Novus). After three additional PBS washes cuts were incubated for 45 minutes at room temperature with secondary antibodies (Dako) followed by three additional washes with PBS and finally mounted in antifade solution containing DAPI (Invitrogen). The expression of ABCG1, SR-BI and EL were compared to a native radial artery frozen in embedding matrix (O.C.T.) and cut as describe above. Analyses of the cuts were performed on an inverted microscope (Zeiss).

### Endothelium integrity

To analyse the endothelial integrity Evan’s blue (Sigma Aldrich) was injected at a final concentration of 0.5% in the circulation loop of the bioreactor for 10 minutes followed by PBS washing phase for 10 minutes. Grafts were cut open and analysed macroscopically with photo documentation.

### Nitric oxide measurement

Nitric oxide synthesis was measured as described previously [22]. In brief, following PBS wash 5 mm vessel rings were incubated in assay buffer (50 mM Tris-Cl, pH7.4, 0.25 mM EDTA, 0.3 mM CaCl_2_, 0.25 mM DTT, 25 µM L-arginine, 1 mM NADP, 100 nM calmodulin, 10 µM tetrahydrobiopterin and 1.25 µCi L-^3^H-arginine; PerkinElmer) in the presence of 10 nM acetylcholine (Ach), 0.5 mg/ml HDL or 1 mM L-NG-nitroarginine methyl ester (L-NAME). After 30 minutes at 37°C, the reaction was stopped by adding 1 ml of ice cold Hepes buffer (50 nM HEPES, 5 mM EDTA, pH 5.5). Tissue was homogenized using a tissue homogenizer (Precellys 24, Bertin Technologies) and the supernatant was load onto ion exchange column (Dowex, 50 WX8-20, Sigma) previously equilibrated with stop buffer to separate L-^3^H-citruline from L-^3^H-arginine. Scintillation mix (Ultimate Gold, PerkinElmer) was added to the supernatant and counted using β-counter (Betamatic, Kontron).

### Statistic analysis

Statistical comparisons between different groups were performed using a ANOVA. The data has been obtained from at least 2 different experiments and were graphically represented as mean ± standard deviation of the mean (SEM). P-values of<0.05 were considered statistically significant. All statistical anaylses were performed using GraphPad Prism-5 software.

## Results

### Structure and characteristics of engineering arteries

**Figure 1:**
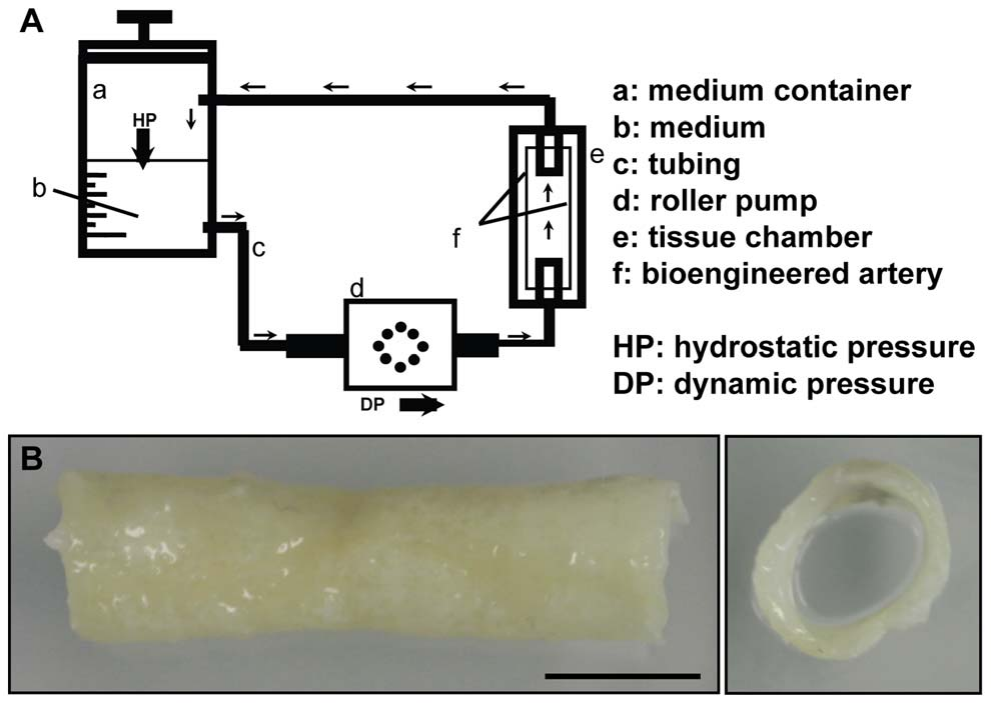
Bioreactor and macrostructure of the engineered artery equivalent. A) Schematic view of the bioreactor set up. B) Macroscopic picture of the bioengineered artery demonstrated the presence of an open lumen after 5 weeks in culture as well as the formation of tissue on the luminal side of the graft. Bar represents 1 cm.

For the assessment on the structural level, the engineered artery equivalents were analysed macroscopically and microscopically. Macroscopically the tube-like scaffolds had dimensions of 4 cm length and about 0.6 cm diameter before seeding. After the in vitro culture for about 5 weeks the scaffold was densely covered by cells and demonstrated a patent lumen (Fig. 1). Some of the grafts (15%) showed a central decrease of the luminal diameter. These grafts were excluded from any further analyses.

**Figure 2:**
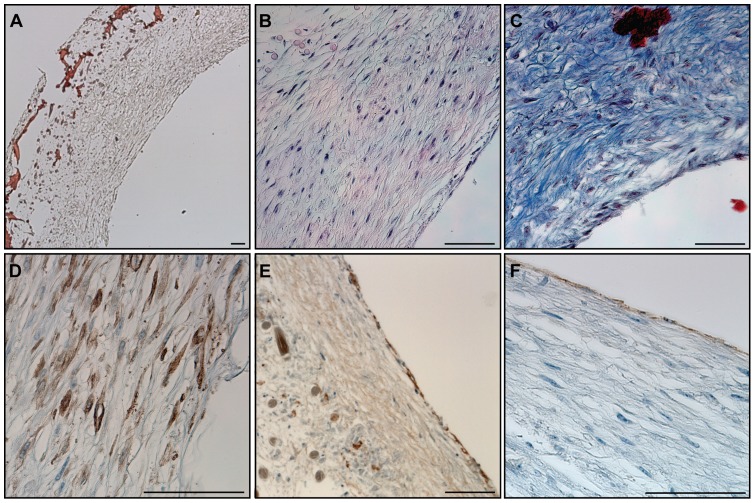
Histological structure of an engineered artery equivalent. A) Haematoxylin-Sudan staining demonstrated the formation of tissue in and on the surface of a PGA/P4HB scaffold as well as the scaffold’s partial degradation. B) H&E staining revealed dense tissue formation composed of cells and extracellular matrix. C) The secretion of collagen was observed after Masson’s trichrome staining. The expression of α-smooth muscle actin (α-SMA) (D) confirmed the smooth muscle phenotype of the cells in the inner layer. Collagen IV positive staining (E) demonstrated the secretion of basement membrane and CD31 positive staining (F) confirmed the presence of an endothelial cell monolayer on the luminal side of the bioengineered artery equivalent. Bars represent 100 µm.

Microscopy analyses with Haematoxylin-Sudan and Haematoxylin-Eosin staining demonstrated the formation of a dense and homogenous tissue on the luminal side of the grafts and a loser tissue formation in contact with the degraded scaffold on the outer surface (Fig. 2 A-B). The tissue was composed of cells and extracellular matrix as demonstrated by the presence of collagen in Masson’s trichrome staining (Fig. 2 C). Immunohistochemistry staining showed the presence of α-smooth muscle actin positive cells in the inner layer of the vessel and CD31 positive cells forming a monolayer on the luminal side (Fig. 2 D-E). Moreover, the presence of a basement membrane underneath the endothelial cells was demonstrated by the presence of collagen IV positive layer (Fig. 2 F).

**Figure 3:**
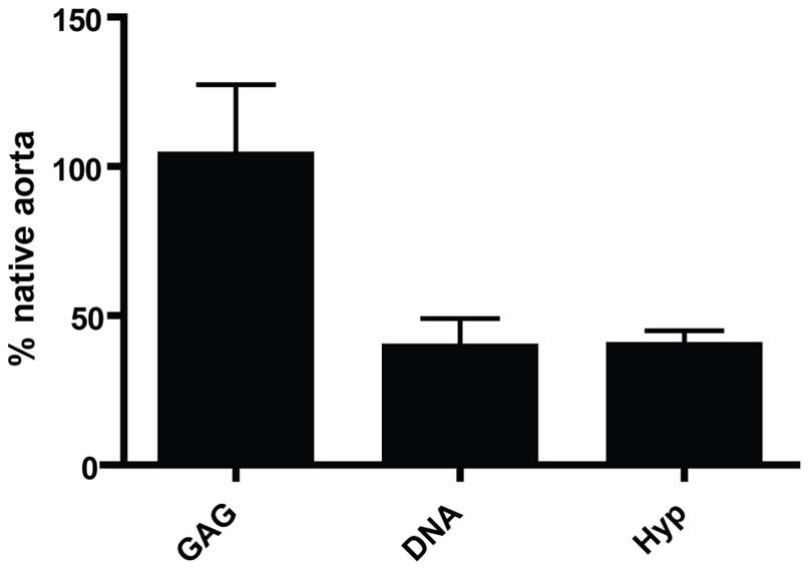
Quantification of extracellular matrix composition and cell number. Total cell numbers, represented as DNA and total collagen secretion of collagen, represented as hydroxyproline (HYP) represented ≈ 50% of the native human aorta. The glycosaminoglycan (GAG) content was similar to native aorta.

The extracellular matrix composition of the graft was further examined with biochemical analyses of the presence of DNA, hydroxylproline (Hyp) or glycosaminoglycans (GAG) and the results were compared with the data obtained for a native human aorta. The GAG composition of the grafts was similar to the values observed in native tissues, 103 ± 23%. Interestingly the DNA and Hyp values represented only 39.5 ± 10% and 40 ± 5%, respectively of the native tissue values (see Fig. 3). In summary, these results demonstrated that the engineered artery equivalents mimicked the cellular structures as well as extracellular matrix composition of a native artery.

### Assessment of endothelial integrity and functionality

**Figure 4:**
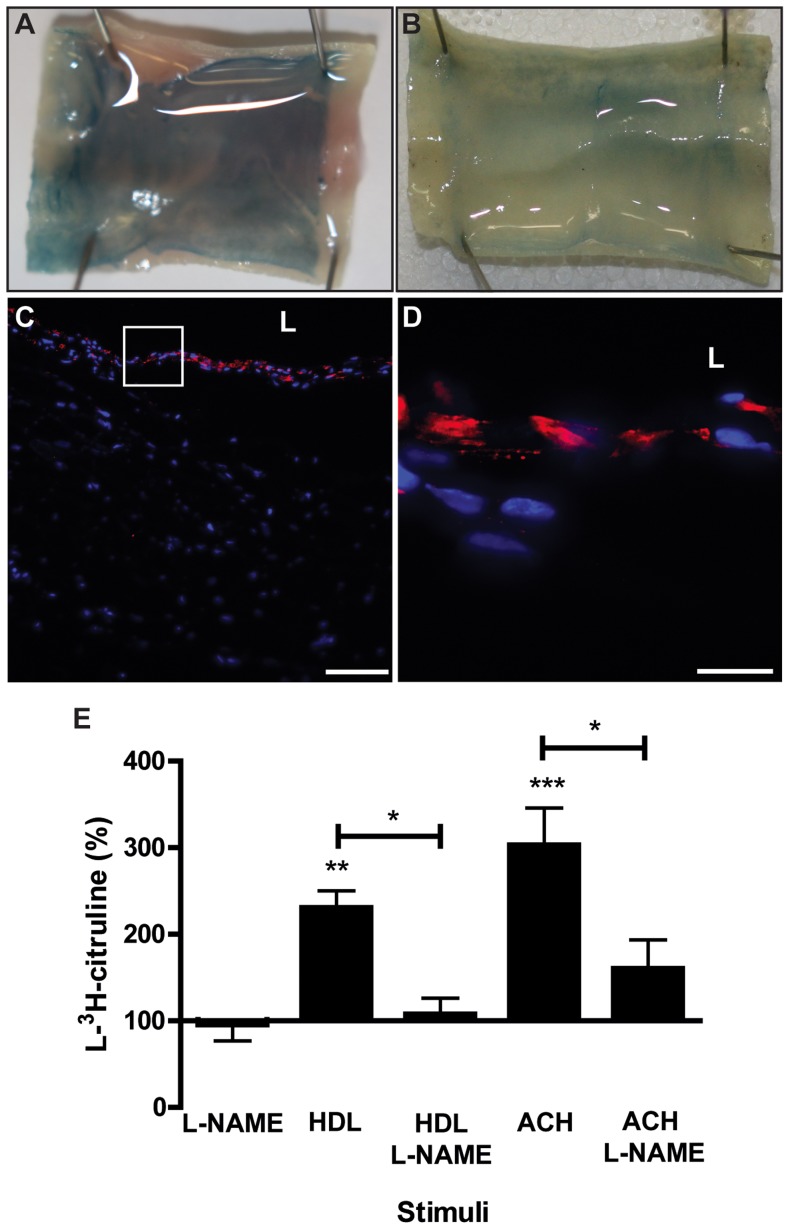
Integrity and functionality of endothelial cells in the engineered artery. The endothelial integrity was analysed using Evan’s blue staining (0.5% for 10 minutes). In the absence of endothelial cell (A) the tissue appeared evenly stained in blue. Two weeks after endothelialization (B) the endothelial barrier retained the absorption of Evan’s blue and tissue appeared non-coloured. The integrity of the endothelium was further analysed by microscopy of cryosections for the expression of the tight junction protein (ZO)-1 (red) by endothelial cells (nuclei: blue). (C & D). Nitric oxide was measured using L-^3^H-arginine as the substrate and in presence of 10 nM acetylcholine (Ach) or 0.5 mg/ml HDL as the stimulator or 1 mM L-NAME as the inhibitor of endothelial nitric oxide synthase (eNOS). After 30 minutes L-^3^H-citruline was separated from L-^3^H-arginine by ion exchange column. Bar represents 100 (C) and 25 µm (D) and L: Lumen. *** p<0.001, ** p<0.01 and * p<0.05

Next, the integrity of the endothelium was analysed using Evan’s blue staining. As expected before endothelialisation, the Evan’s blue dye was penetrating into the tissue and colouring evenly the bioengineered graft. (Fig. 4 A). One week after the seeding of endothelial cells, the subendothelial was less intensively stained with Evan’s Blue, indicating the beginning but not complete endothelialisation (data not shown). Two weeks after endothelial cell seeding, the tissue was not coloured by Evan’s blue any more indicating the presence of a tight endothelium (Fig. 4 B). To confirm the presence of tight junctions between the endothelial cells cryopreserved tissue was stained for tight junction protein 1 (ZO-1). The presence of ZO-1 expression on the luminal side of the graft was demonstrated two weeks after endothelial cell seeding (Fig. 4 C and D). To further characterize endothelial properties, we measured nitric oxide (NO) production by the grafts. After incubating two weeks old endotheliazed vessels with 0.5 mg/ml HDL (p<0.01) or 10 nM acetylcholine (p<0.001) we measured a significant increase of L-^3^H-arginine to L-^3^H-citruline and NO conversion. In the presence of the specific NO synthase inhibitor L-NG-nitroarginine methyl ester (L-NAME), the conversion was significantly reduced (p<0.05) indicating NO synthesis by endothelial cells in the grafts after both HDL and Ach stimulation (Fig 4 E). Taken together these data suggest that the endothelium of the engineered artery equivalent formed a functional barrier isolating the subendothelial tissue from the luminal fluid as shown with the Evan’s blue assay.

### Low-density lipoprotein insudation and accumulation in the tissue

**Figure 5:**
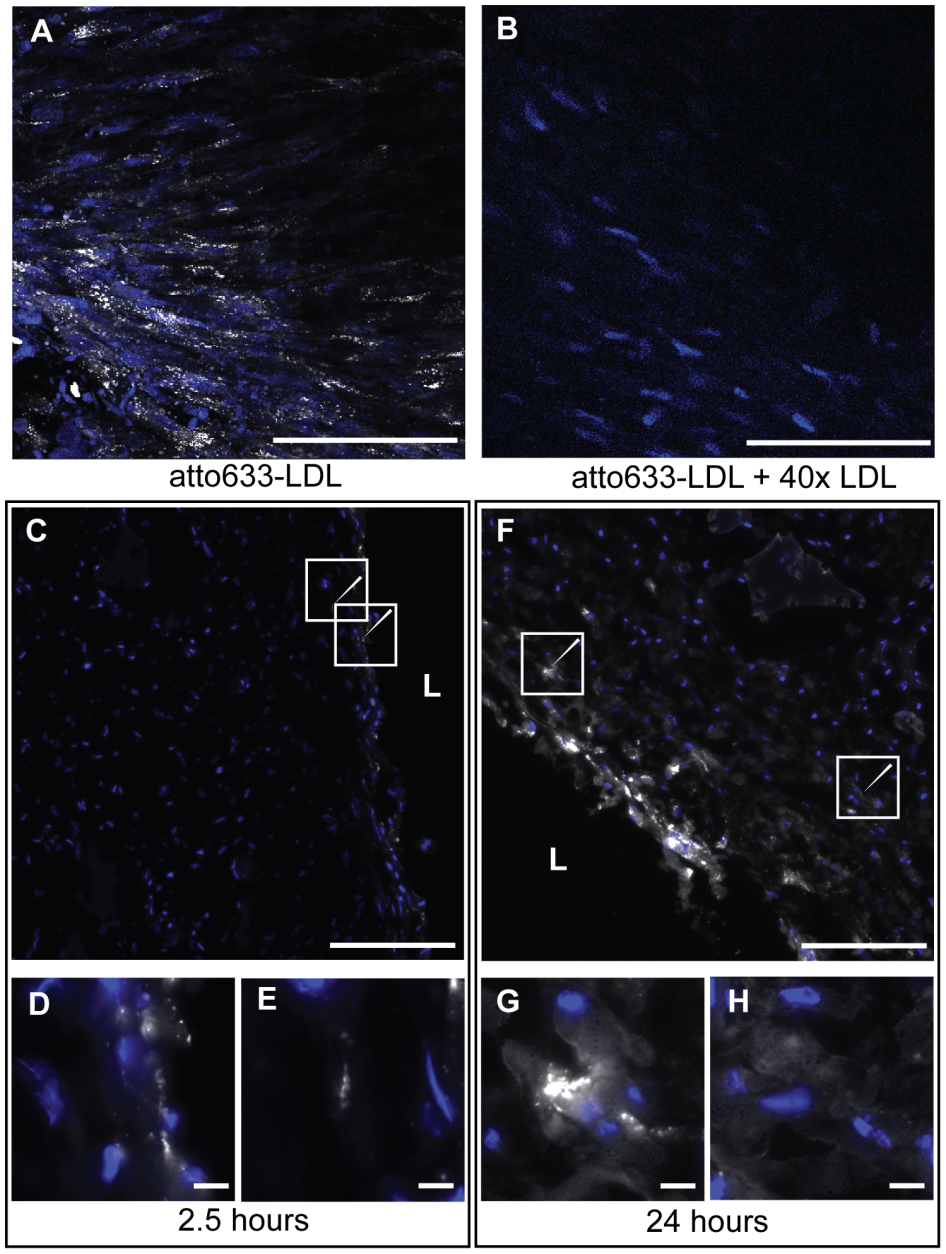
Insudation of LDL into the engineered artery. The insudation of LDL into the tissue (blue: nuclei) was analysed by confocal microscopy after incubation with 20 µg/ml of fluorescent LDL (white) for 24 hours and demonstrated the vesicular localization on the upper cell layer (A). The specificity of the signal was assessed by competition with 40-fold excess of non-labelled LDL (B). LDL localization in the tissue was further analysed by microscopy of tissue cryosections and demonstrated the time-dependent uptake and the sub-endothelial localization of the LDL (C: 2.5 hours and F: 24 hours). Zoom in of interesting regions shown with the arrows are presented in D and E for 2.5 hours and F and G for 24 hours. Bars represent 200 µm (A-C,F) and 20 µm (D-E, G-H) and L: Lumen.

The insudation and accumulation of LDL into the vasculature represent early events in atherosclerosis development. To investigate whether our engineered artery equivalent is suitable to mimic this step in atherogenesis, fluorescently labelled LDL was injected into the circulation loop of the dynamic graft culture. The tissue was collected after 2,5 and 24 hours and analysed by confocal imaging after fixation or cryopreserved for immunofluorescence. Confocal images were taken from the lumen of the graft and then – by changing the z-plane – by focusing deeper into the tissue. LDL was clearly localized intracellular in cytosolic vesicles within the luminal cell layer after 24 hours (Fig. 5 A). To verify the specific uptake of LDL, the engineered grafts were incubated with fluorescently labelled LDL in the presence of a 40 fold excess of non-labelled LDL, the signal was dramatically reduced (Fig. 5 B). This observation confirms the specificity of the LDL up-take into the engineered artery equivalent. Cryo-sections of the graft isolated at 2.5 and 24 hours demonstrated a time dependent uptake of LDL by the tissue. Indeed the signal emitted by the fluorescent LDL increased over time (Fig. 5 C & F). After 2.5 hours, LDL was mainly localized within the endothelial cell layer with minimal signal in the sub-endothelial space (white arrow Figure 5 C-E). After 24 hours LDL was still localized in the endothelial cell layer but the sub-endothelial tissue revealed an increase of fluorescence (white arrow Fig. 5 F). Indeed in the sub-endothelial tissue LDL was found to accumulate either in spots (Fig. 5 G) or diffuse areas (Fig. 5 H). These results indicate the possibility to study trans-endothelial transport and sub-endothelial accumulation of LDL in our model.

### Monocyte binding and insudation in engineering artery

**Figure 6:**
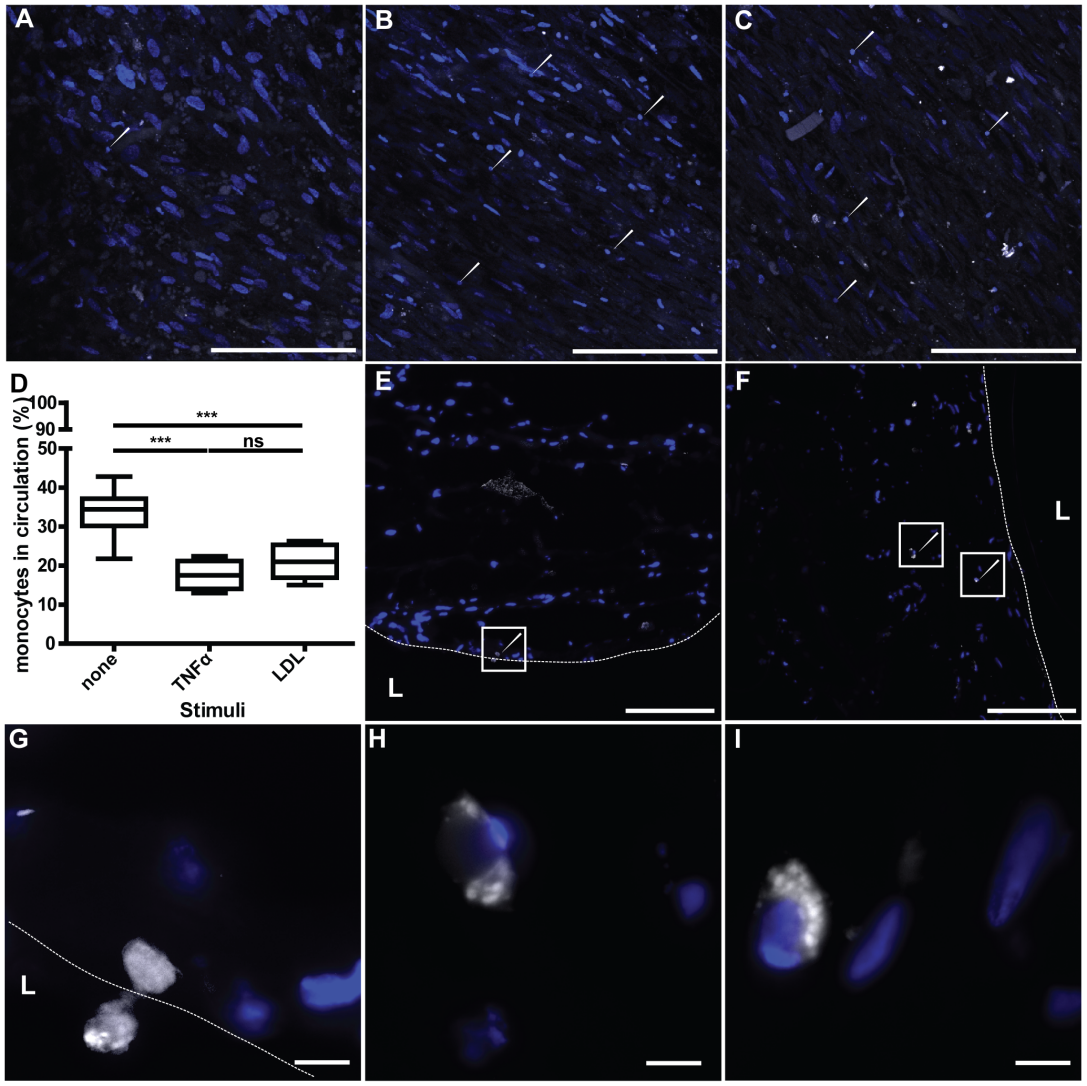
Endothelial monocyte adhesion and transmigration migration in the engineered artery. After pre-treatment in the absence (A, D) or the presence of 3 hours TNFα (10 ng/ml) (B, D) or 24 hours LDL (20 µg/ml) (C-I) 1×10^6^ fluorescently labelled monocytes (white) per ml were injected into the circulation loop and circulated for 24 hours. Tissues were analyzed by confocal microscopy and after cryosectionning. In addition, monocytes remaining in the circulation were counted. More monocytes (white arrows) adhered after pre-treatment with TNFα (B) or LDL (C) compared to the not stimulated (A). Less monocyte remained into the circulation after TNFα or LDL pre-treatment compared to the absence of stimuli (D). Monocytes adhesion and migration in the tissue was further analyzed by microscopy of cryosections after LDL pre-treatment. Microscopic observations demonstrated adhesion and migration of monocytes through the endothelium (dash line) (E, G) and accumulation of monocytes into the tissue (F, H-I). Bars represent 200 µm (A-C, E-F) and 20 µm (G-I).

To investigate if our model is suitable to analyse the attachment and trans-endothelium migration of monocytes into the interstitial vascular tissue, labelled monocytes were injected into the bioreactor circulation loop and tracked by confocal imaging and cryo-section analysis. The isolated monocytes were CD14 positive and after in vitro culture they express CD68 (data not shown). Monocytes were labelled with SNARF-1 dye after the isolation and the capacity of the labelled monocytes to bind to activated endothelial cells was verified in conventional cell culture (data not shown). The labelled monocytes were injected into the circulating medium of the graft in the absence or presence of 3 hours pre-treatment with TNFα. After 24 hours incubation, the attachment of the monocytes on the arterial graft was monitored by confocal imaging and demonstrated an increased number of monocytes adhering to the graft after TNFα pre-incubation (white arrows; Fig. 6 A-B). Moreover after 24 hours pre-incubation of the graft with LDL, the monocytes attached to the endothelium to a similar extent as compared with the TNFα pre-stimulation (Fig. 6 C). Interestingly, if the monocytes were added simultaneously with the LDL no increase of monocyte adhesion could be observed compared to the non-stimulated grafts (data not shown). In addition, the attachment of the monocytes to the scaffold alone was investigated and demonstrated no adhesion. To quantify monocyte adhesion, monocytes having remained in the circulation after 24 hours were counted and compared to the monocyte number of the control group (monocyte perfusion of scaffold only). The numbers of monocytes in the circulation after TNFα and LDL pre-stimulation 17.5±3.0% and 20.8±3.5% respectively were not statically different (p = 0.226). However, they were significantly lower compared to the not stimulated engineered arteries (33,7±2,8%), p<0.0001 for TNFα and p<0.0001 for LDL. These results indicate that significantly more monocytes were bound under stimulated conditions. Microscopy of the cryo-sectioned tissue after LDL pre-incubation demonstrated the presence of monocytes adhering to the endothelial surface and migrating into the tissue (Fig. 6 E & G). In addition, monocytes were also identified in the tissue after LDL pre-treatment (Fig. 6 F & H-I). These results indicate that our engineered artery equivalent represents a suitable tool to study the attachment and transmigration of monocytes into the artery, which represents a key process in the development of atherosclerosis in vivo.

### High density lipoprotein insudation in engineered artery

**Figure 7:**
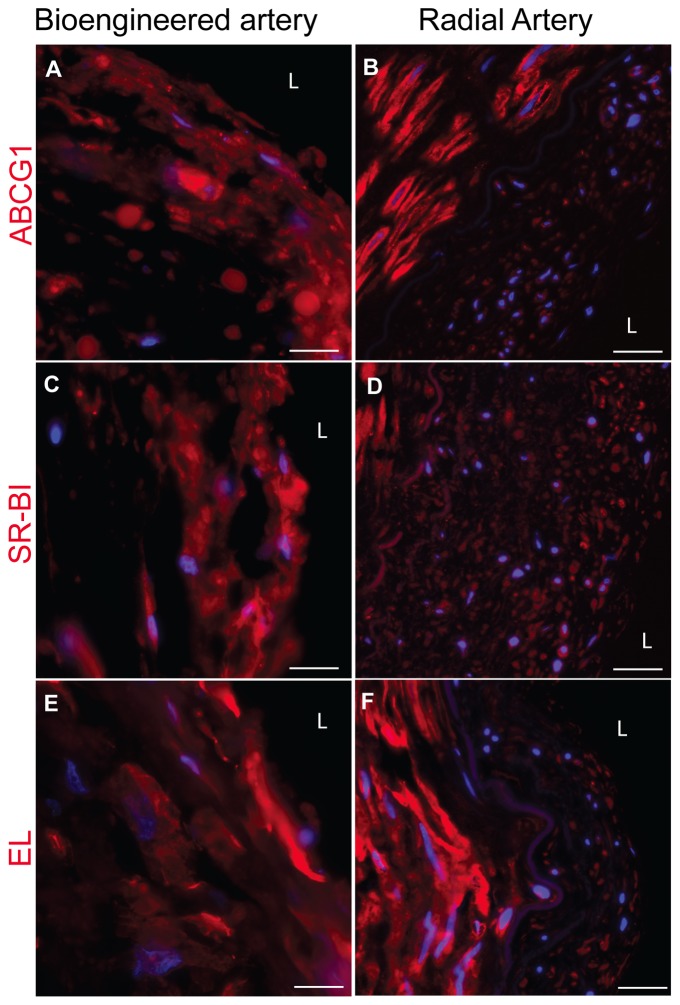
Expression of ABCG1, SR-BI and EL in the bioengineered artery in comparison to native artery. The expression of ABCG1 (red) (A), SR-BI (red) (C) and EL (red) (E) was assessed by immunofluorescence staining after cryosection of the engineered artery and compared to a native radial artery (B, D, F) (blue: nuclei). Bars represent 50 µm.

**Figure 8:**
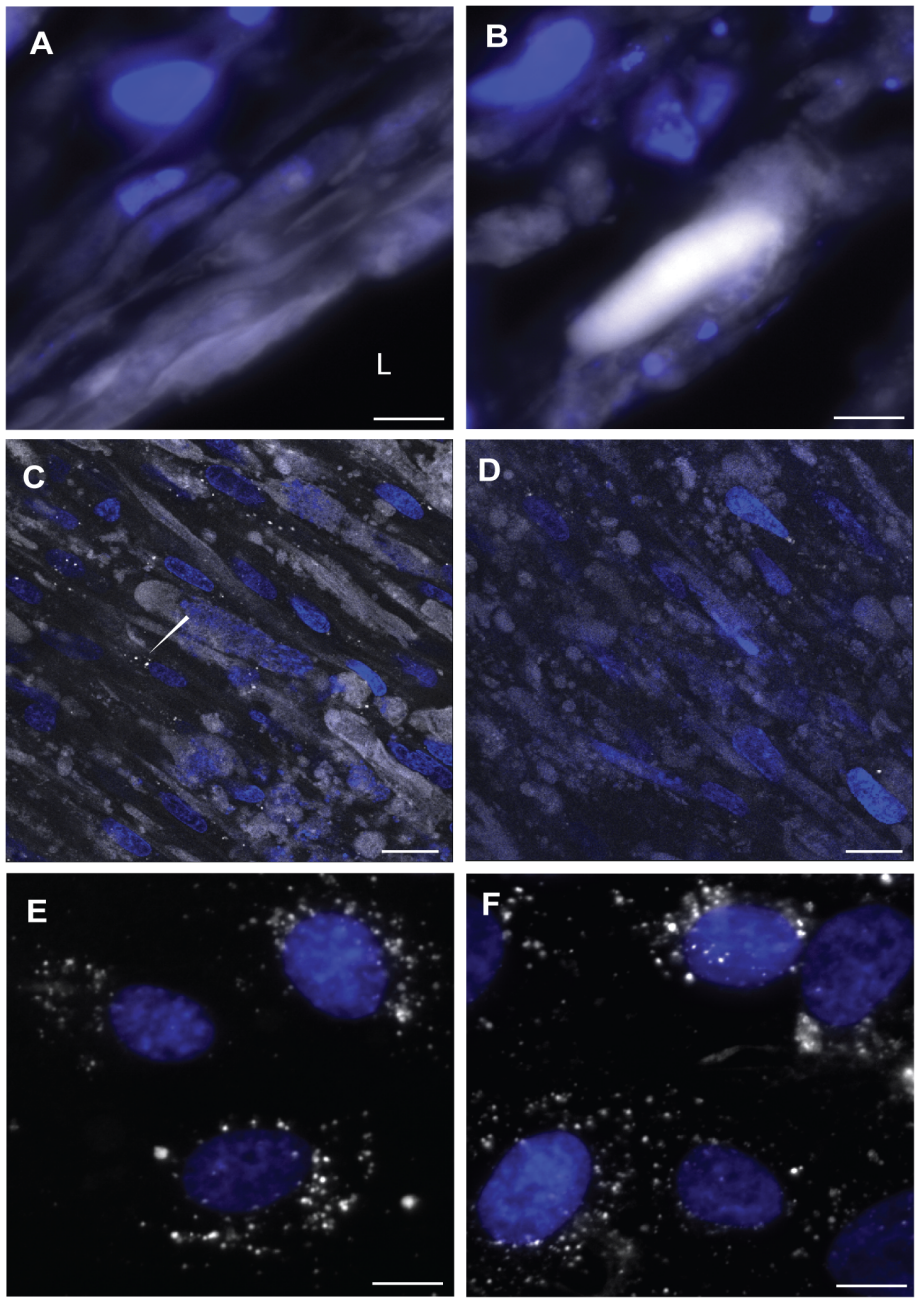
HDL insudation in the engineered artery equivalent. 25 µg/ml of HDL was circulated for 24 hours. The insudation of HDL (white) was assessed after cryosection of the tissue on the luminal side (A) and in the tissue (B) (blue: nuclei). HDL localization in the tissue was further analysed by confocal microscopy at 2.5 um (C) and 16 um (D) deep in the tissue. The arrow shows the intracellular vesicular localisation. The up-take of HDL (white) into HUVECs (E) and UCMFB (F) was analysed in regular cell culture after 24 hours incubation with 25 µg/ml. Bars represent 50 µm and L: Lumen.

HDL is an important key player in the development of atherosclerosis development. Indeed HDL is believed to have a major anti-atherosclerotic potential by removing the excess cholesterol out of the macrophages in the arterial wall. In this context, the trans-endothelial transport of HDL remains a potentially limiting step to be elucidated [23]. The ATP binding cassette (ABC) G1, the scavenger receptor (SR)-BI and endothelial lipase (EL) were demonstrated to participate in the trans-endothelial transport of HDL in cell culture [24]. In order to analyse HDL transport across the endothelium the expression of the known HDL binding proteins were investigated by immunofluorescence stainings. Specific stainings with antibodies against ABCG1, SR-BI and EL demonstrated the high expression of those markers by both HUVECs and HUMFBs in the engineered artery equivalent in a comparable pattern as in a native artery (Fig. 7). To further investigate whether our model is suitable to analyse the transport of HDL through endothelial cells, we incubated the bioengineered graft with fluorescently labelled HDL. Similar as described for LDL, cryosection of the harvested engineered arteries demonstrated the presence of HDL in the endothelial cells as well as the accumulation of HDL in the sub-endothelial space (Fig. 8A). In addition, the distribution of HDL was analysed by confocal microscopy. Interestingly, HDLs were localised either in intracellular vesicles or as extracellular diffused material if incubated with engineered arteries under flow conditions (Fig. 8B). However, under static non-flow conditions labelled HDL was recovered in intracellular vesicles only (Fig. 8 C & D). These differences between static (non-flow) cell culture conditions and the flow conditions of our engineered arteries demonstrates the potential of the proposed model to study HDL transport under in vitro in conditions which are closer to the native situation.

## Discussion

Given its substantial negative socioeconomic impact, atherosclerosis has been intensively studied in the last decades. In particular, many efforts have been undertaken to develop an adequate ex vivo model for the biofunctional large-scale assessment of ethiopathogenetic phenomena in vitro. The development of endothelial and smooth muscle cell co-culture systems for the assessment of monocyte transmigration and accumulation in the artery intima, representing a key step in the pathogenesis of atherosclerosis, was already proposed in the early 1990’s [5], [6]. However, as already emphasized by Dorweiler et al., these co-culture systems allow neither for long term in vitro culture nor histology and extracellular matrix analyses [8]. Their own co-culture system on a fibrin gel allowed longer culture times for up to 6 weeks and hence represented a major step forwards the in vitro modelling of atherosclerosis[8], [9]. Nevertheless, in spite of this improvement, also this approach was missing a major physiologic aspect evident in the biology of native arteries - that is the presence of (pulsatile) blood flow in a lumen. This seems most important because atherosclerotic lesions prevail at the site of altered blood flow as first reported by Caro et al. [25].

Therefore, the present study addressed the question whether bioengineered artery equivalents - as previously developed as therapeutic replacements for patients with structural cardiovascular disease [10]–[13]- are suitable for modelling essential pathogenic phenomena of atherosclerosis in vitro. In particular, the pulsatile native-like flow environment could represent a major advantage in this regard when compared to previous studies using static cell culture environments.

As previously shown, histological analyses of the bioengineered grafts demonstrated a structure similar to the layered microstructure of the native artery [10], [14], [26]. These constructs were equally covered with a multi-layer of α-smooth muscle actin positive cells indicating the development of a media-like layer. The presence of secreted extracellular matrix components, such as collagen and glycosaminglycans demonstrated the in situ functionality of smooth muscle cells in the tissue. However, the biochemical DNA and the collagen quantification, revealed lower values than found in the native artery. This phenomenon has been previously described and supposed to be due to the relatively short in vitro culture periods [10]. This is further supported by the experiments in large animal models, where tissue engineered arteries were exposed to longer in vitro and in vivo conditioning and eventually showed higher extracellular matrix values [14]. The positive layer of collagen IV suggested the presence of a basement membrane, that is secreted by the endothelial cells and separates them from the subendothelial space [27]. In addition, the luminal side was covered with a monolayer of endothelial cells on top of the basement membrane. Another major function of the endothelium is the formation of a tight barrier between the blood and the interstitial vascular tissue. In our engineered grafts, the integrity of the endothelial layer was confirmed by its impermeability for Evan’s blue stain as well as by the expression of the ZO-1. Endothelial functionalty was confirmed by NO production and monocyte adhesion after TNFα stimulation.

The microscopic demonstration of labelled LDL in intracellular vesicles and the sub-endothelial space indicate the uptake and resecretion of LDL. According to the response-to retention model, the accumulation of LDL in the sub-endothelial space is a crucial step in the formation of atherosclerotic lesions [28]. Besides the accumulation of LDL, inflammatory processes play an indispensable role in the pathogenesis of atherosclerosis, for example by endothelial cell activation and subsequent monocyte adhesion [29]. Therefore, we applied TNFα to analyse monocyte adhesion to the endothelium. The observed significant increase of monocyte adhesion after stimulation with either TNFα or LDL hence provides another indication that our model is feasible to study pathogenic events.

The trans-endothelial transport of HDL in the arterial wall for removal of cholesterol accumulation is a key step in the reverse cholesterol transport [3]. In addition, apoA-I - the major apolipoprotein of HDL - was found to be present in atherosclerotic plaque by recent investigations [30]. In agreement with these histological descriptions and our previous findings of trans-endothelial HDL transport in transwell cell culture we found immunofluosecently labelled HDL both in endothelial cells and the sub-endothelial space of our 3D model.

In conclusion, we demonstrate for the first time the feasibility to engineer a dynamic 3D human artery equivalent for the investigation of fundamental processes involved in the development of atherosclerosis in vitro. In the contrary to previous investigations, this model combines the engineering of a native-like multilayer 3D architecture of the vascular wall with a lumen and the applicability of native-analogous pulsatile flow profiles. Thereby, this unique model mimics the native-like vascular environment and may therefore also allow for more representative in vitro investigations with higher predictive value for human pathologies in vivo. In addition, this model may also allow for large scale in vitro drug screening experiments – in particular by enabling the specific modulation of several parameters (i.e. flow, pressure, LDL, HDL, type of immune cells, etc). Thus, this model may ultimately help to significantly reduce the necessity of animal experiments in the field of atherosclerosis research. We therefore believe that our model represents an important step towards the development of a native-like large-scale atherosclerosis in vitro disease model, which may help to improve our understanding of this important pathology.
